# Bioelectricity Generation in a Microbial Fuel Cell with a Self-Sustainable Photocathode

**DOI:** 10.1155/2015/864568

**Published:** 2015-04-30

**Authors:** Ting Liu, Liqun Rao, Yong Yuan, Li Zhuang

**Affiliations:** ^1^Orient Science & Technology College, Hunan Agricultural University, Changsha 410128, China; ^2^Guangdong Institute of Eco-Environmental and Soil Sciences, Guangzhou 510650, China; ^3^College of Bioscience and Biotechnology, Hunan Agricultural University, Changsha 410128, China

## Abstract

This study aims to construct an MFC with a photosynthetic algae cathode, which is maintained by self-capturing CO_2_ released from the anode and utilizing solar energy as energy input. With this system, a maximum power density of 187 mW/m^2^ is generated when the anode off gas is piped into the catholyte under light illumination, which is higher than that of 21 mW/m^2^ in the dark, demonstrating the vital contribution of the algal photosynthesis. However, an unexpected maximum power density of 146 mW/m^2^ is achieved when the anode off gas is not piped into the catholyte. Measurements of cathodic microenvironments reveal that algal photosynthesis still takes place for oxygen production under this condition, suggesting the occurrence of CO_2_ crossover from anode to cathode through the Nafion membrane. The results of this study provide further understanding of the algae-based microbial carbon capture cell (MCC) and are helpful in improving MCC performance.

## 1. Introduction

Microbial fuel cells (MFCs) are devices that convert organic waste material into electricity energy by using microorganisms as biocatalysts. The environmentally friendly process has been gaining international attention in recent years as an advanced technology for both electricity generation and waste treatment [1–3]. Common MFC is a dual-chamber system, consisting of an anode and a cathode chamber that is separated by a proton exchange membrane (PEM) [4]. In such a system, oxygen must be continuously supplied for the reaction in the cathode, leading to extra energy consumption for aeration [5]. Therefore, requiring continuous aeration is obviously a limitation for real-world applications of MFCs because of its economic and environmental cost.

As a solution to eliminate or maintain minimum energy consumption for cathode aeration, recently studies have proposed the integration of algal photosynthesis with MFCs, which was known as photo MFCs [6–8]. In such a system, oxygen was produced* in situ* in the cathode compartment through the algal photosynthesis. It is known that algae are responsible for ca. 75% of the earth's oxygen production during their uptake of carbon dioxide (CO_2_) under solar light illumination [9, 10]. Therefore, the algae-based photo MFCs are capable of simultaneously fixing CO_2_, generating electric energy, and treating wastewater, representing a more advanced technique as compared with the conventional MFCs [11–13]. In these cases, CO_2_ was required to be continuously supplied to the cathode compartment to maintain high energy generation. Similar to aeration, CO_2_ purging consumed extra energy, representing an unsustainable option in terms of economy. In this regard, Wang et al. [14] demonstrated a new microbial carbon capture cell (MCC) in which CO_2_ generated from substrate degradation in the anode chamber was directly introduced to the cathode for the O_2_ production via algal photosynthesis. This MCC represents an effective technology for simultaneous CO_2_ emission reduction and voltage output without aeration. In spite of the advantages, this system is still in its infancy stage. To date, there are still very few attempts regarding how MCCs will work in a more sustainable manner.

The objective of this study was to fully understand MCC systems in terms of electric energy production performance and measure the microenvironments of the photocathode under various circumstances to reveal the underlying mechanisms of cathode reactions. For this purpose we constructed an MCC system and examined its power generation process under light-on and light-off circumstances. Variations in pH and dissolved oxygen in the catholyte were monitored* in situ* with microelectrodes during the circumstances. In addition, conventional electrochemical techniques were used to reveal the catalytic activities of both anode and cathode. Scanning electron microscopy was employed to explore the morphology of biofilms formed on both the anode and the cathode. Generation of bioelectricity by the MCC was related to pH and oxygen variations at the cathode. The results from this study are expected to be helpful in further improving MCC performance.

## 2. Materials and Methods

### 2.1. MCCs Setup and Operation

**Figure 1:**
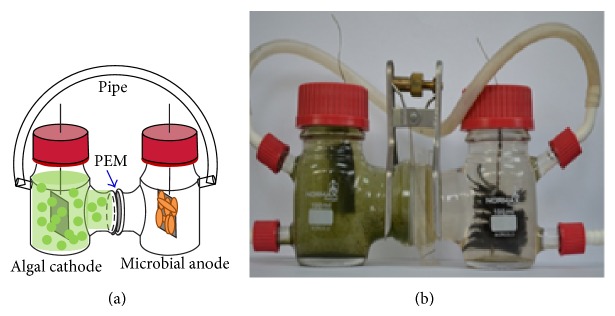
Schematic diagram (a) and experimental setup (b) of an MCC. CO_2_ is transported through a silicon tube, fixed on the chambers.

The MCCs consisted of two 100 mL glass bottles as two chambers separated by a piece of PEM as shown in Figure 1. Gas generated from the anode was piped into the catholyte. The anode was made of carbon fiber brushes and the cathode was carbon felt (3 cm × 3 cm) containing 0.1 mg/cm^2^ Pt catalyst (10%, Pt loading, HESEN, China). To coat the Pt catalyst to the carbon felt, Pt/C powder was mixed with Nafion solution and then applied to the carbon felt surface with a brush. Both electrodes were connected with titanium wire. A white light LED (light intensity of 2000 lx) was employed to continuously illuminate the cathode. When operated in the dark, the cathode chamber was wrapped with an aluminium foil. All experiments were conducted in duplicate.

The MCC anode chamber was inoculated with 10 mL of activated anaerobic sludge (Liede Sewage Treatment Plant, Guangzhou, China) and 90 mL of culture medium (pH = 7.0). The anode culture medium contained sodium acetate (1,000 mg L^−1^), NaH_2_PO_4_·2H_2_O (2.77 g L^−1^), Na_2_HPO_4_·12H_2_O (11.40 g L^−1^), NH_4_Cl (0.31 g L^−1^), KCl (0.13 g L^−1^), a vitamin stock solution (12.5 mL L^−1^), and a mineral stock solution (12.5 mL L^−1^) [15]. The acetate-free phosphate buffered solution was used as cathode medium. To start up the MCC, the cathode was firstly purged with oxygen. After the voltage output was stabilized,* Chlorella vulgaris* was introduced into the cathode chamber and the LED lamp was turned on to illuminate the cathode chamber.* C. vulgaris* was purchased from FACHB-Collection (FACHB 1068, China) and cultured in an illuminated autoclaved flask aerated with air. The algae suspension was centrifuged (10,000 ×g) and washed 3 times with DI water before added to the cathode compartment. Then the cathode chamber was purged with N_2_ to exclude the influences of the remained oxygen before piping CO_2_. The power density curves of the MCC were obtained by changing the circuit resistor from 10,000 to 50 Ω. All tests were conducted in batch mode in a 30°C incubator. The cell voltage was recorded every 2 min by a digital multimeter connected to a computer. The power was normalized by the projected surface area of the cathode.

### 2.2. Microelectrode Measurements

The cathode pH measurements were conducted continuously using a pH microsensor (50 *μ*m in diameter, response time of ca. 30 s) connected to a multimeter (Unisense, Aarhus, Denmark) for 60 hours. The sensor was calibrated using standard pH buffers before use. Oxygen was measured incessantly using an oxygen microsensor (50 *μ*m in diameter, response time of ca. 5 s) connected to the same equipment for 60 hours. Before taking measurements, the oxygen microsensor was polarized at +800 mV to achieve a stable signal output. The sensor was calibrated in both oxygen-saturated and oxygen-free solutions.

### 2.3. Analytical Techniques

Cyclic voltammograms (CVs) were conducted in a conventional three-electrode electrochemical system by a potentiostat (CHI660D, Chenhua Instrument, China). A saturated calomel electrode (SCE) was used as reference electrode. The anode and cathode of the MCC were used as working and counter electrode, respectively. The linear sweep voltammetry (LSV) of the cathode was measured with potentials ranging from +0.4 V to −0.7 V (versus SCE) by the potentiostat. Sodium acetate concentration was determined by HPLC (Waters1525, Binary HPLC Pump). Samples were filtered (0.2 *μ*m filter) before HPLC analyses using an Agilent Zorbax SB-C18 (250 × 4.6 mm, 5 *μ*m) column, with 0.01 mol L^−1^ phosphate buffer as the mobile phase (1.0 mL/min). Scanning electron microscopy (SEM) was used to study the morphologies of the cathode algae and the anode bacteria, respectively. Briefly, biofilms formed on the anode and the cathode were fixed directly with glutaraldehyde (2.5%, final) for 5 h. Furthermore, the biofilms were washed and dehydrated by successive 30 min incubations in 25% ethanol, 50% ethanol, 70% ethanol, and 100% ethanol. After dehydration, the biofilms were dried with a critical-point dryer (HCP-2, Hitachi, Japan). The same treatment was conducted for the cathode. The specimens were observed by SEM (JEOL, JSM-6330F, Japan).

## 3. Results and Discussion

### 3.1. Power Generation of the MCC in Response to Light Illumination

**Figure 2:**
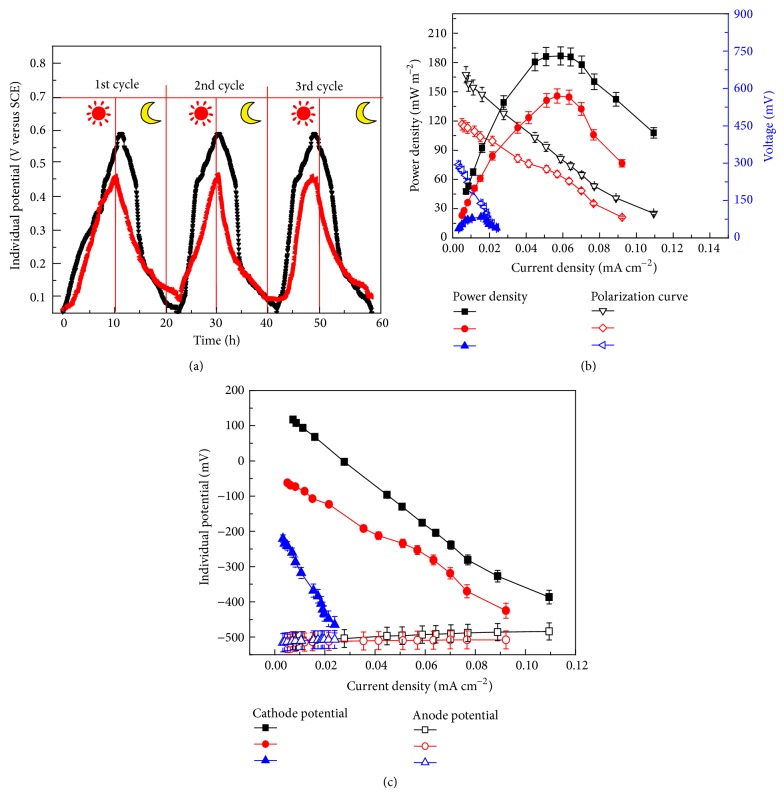
(a) Potential changes of the MCCs under different circumstances (black line is when CO_2_ travelled through the silicon tube and red line is when CO_2_ travel through the silicon tube was blocked with a clip. The symbols of moon and sun represent dark and light conditions, resp.). (b) Power density and polarized curves of the MCCs operated under various conditions. (c) Individual cathode and anode potentials versus current density curves (black line: CO_2_ travelled through the tube under light illumination; red line: CO_2_ travel through the silicon tube was blocked under light illumination; blue line: CO_2_ travelled through the silicon tube in the dark).

After the MCC was started up and produced stable voltage using an MFC mode with an aerated Pt/C cathode, the anode off gas was piped into the catholyte and* C. vulgaris *was introduced into the cathode compartment. As shown in Figure 2(a), after startup of the MCC, the voltage output from the MCC was significantly affected by illumination. The peak voltage reached 0.60 V under illumination at 2000 Ω. Without aeration in this period, the cathode reaction depended on the O_2_ generated by algal photosynthesis in the cathode compartment. Once the light was turned off, the voltage started to decline, resulting in a final voltage output of 0.1 V in the dark. It is worth mentioning that the uptake of CO_2_ is critical for algal photosynthesis. In this case, CO_2_ generated from the anode was piped into the catholyte to support the algal photosynthesis. The variation of the voltage was consistent with the findings by Xiao et al. [8] who purged the algal cathode with CO_2_ gas. It was expected that no voltage would be produced if the CO_2_ produced in the anode chamber was no longer piped into the cathode. However, considerable although slightly smaller voltage was still generated with the same response on the light.

Furthermore, power densities of the MCCs and individual potentials of electrodes under different operation modes were evaluated and the results were shown in Figures 2(b) and 2(c). When the MCC was continuously illuminated, the maximum power densities were 187 mW/m^2^ (1.7 W/m^3^ by normalizing to the anode volume) and 146 mW/m^2^ (1.3 W/m^3^) when CO_2_ was piped or not piped into the catholyte, respectively. These values were significantly higher than that of 21 mW/m^2^ obtained in the dark. As shown in Figure 2(b), the differences in peak power densities were mainly caused by the performance of cathode. The produced power per anode volume was comparable with the previous results [11, 16], but a little smaller than that reported by Wang et al. [14]. The difference in power density possibly resulted from the different reactor configurations. Here, we used a traditional H-type reactor with easily maintained anaerobic environment for the anode, but such a reactor had a higher internal resistance as compared with that used by Wang et al. [14], resulting from the long distance between the anode and the cathode and the small size of the Nafion membrane [17, 18].

On the other hand, results of previous study showed that light was the most important parameter for MCCs performance. Light dependent performance of the algal photo MFC was also observed by Gouveia et al. [19]. However, a self-sustained sediment phototrophic MFC containing both photosynthetic microorganisms and heterotrophic bacteria was reported to generate a higher power density in darkness than that in the light [20]. The inconsistency of results in the effect of light on MFCs is attributed to the different nature of algae [16].

### 3.2. Electrochemical and Morphological Characteristics of the Electrodes

**Figure 3:**
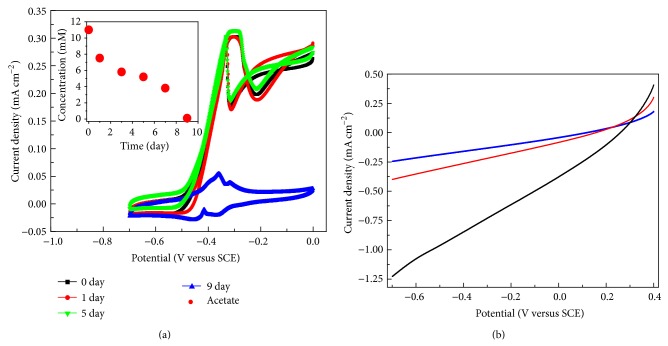
(a) The cyclic voltammograms (CVs) of the anode at different times (inset demonstrates the relationship between time and acetate concentration). (b) The LSV of the cathode under various conditions (black line: CO_2_ travel through the tube was allowed under light illumination; red line: CO_2_ travel through the silicon tube was blocked under light illumination; blue line: CO_2_ was allowed to travel through the tube in the dark).

As mentioned above, the performance of the MCC was mainly limited by the cathode. However, a stable bioanode should be maintained for voltage output during the examined light-on and -off periods. As shown in Figure 3(a), sigmoidal CVs were observed from all anodes for the first 5 days, demonstrating catalytic oxidation of acetate by these bioanodes. At the 9th day, no catalytic current was observed, which suggested the complete consumption of acetate (Figure 3(a), inset). In this case, a nonturnover CV behavior of the bioanode was observed, which showed two major redox couples. The CV was similar to those reported electrochemically active biofilms based on* Geobacter sulfurreducens* [21, 22].

**Figure 4:**
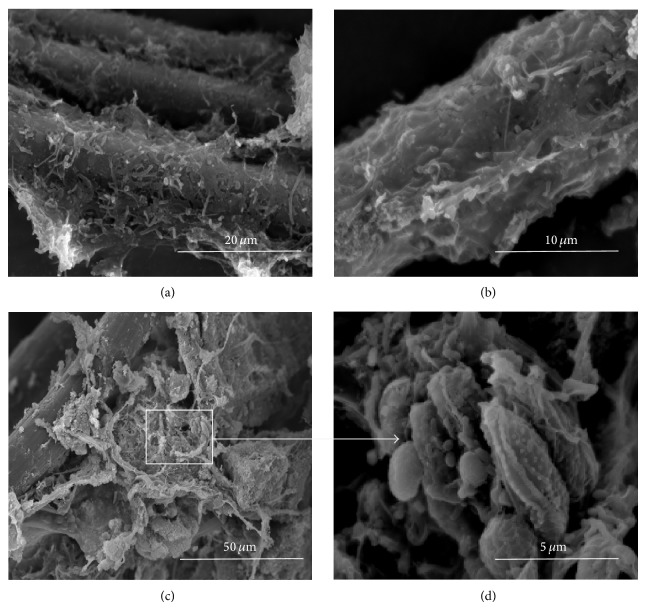
(a) and (b) SEM images of the anode biofilms; (c) and (d) SEM images of the cathode biofilms.

On the other hand, linear sweep voltammetry (LSV) was used to investigate the electrochemical catalytic reaction of the cathode. As shown in Figure 3(b), catalytic current from oxygen reduction was observed when the cathode was piped with the anode off gas and illuminated with light. A decreased catalytic current of the oxygen was observed when the cathode was not piped with the anode off gas, suggesting lower dissolved oxygen in the catholyte under this condition. The catalytic performance of the cathode was well consistent with the voltage and power output as mentioned above, further suggesting the influence of the cathode on the performance of the MCC. After long-term operation, the morphologies of both the anode and the cathode were revealed with SEM. Figures 4(a) and 4(b) showed the present of clustered bacteria on the anode, suggesting formation of electrochemically active biofilm. Similarly, biofilm was also formed on the cathode electrode, demonstrating that algal cells were also possible to adsorb on the surface of the cathode as shown in Figure 4(c). Figure 4(d) showed the algal cells were in round-shape.

### 3.3. The Cathodic Microenvironments

**Figure 5:**
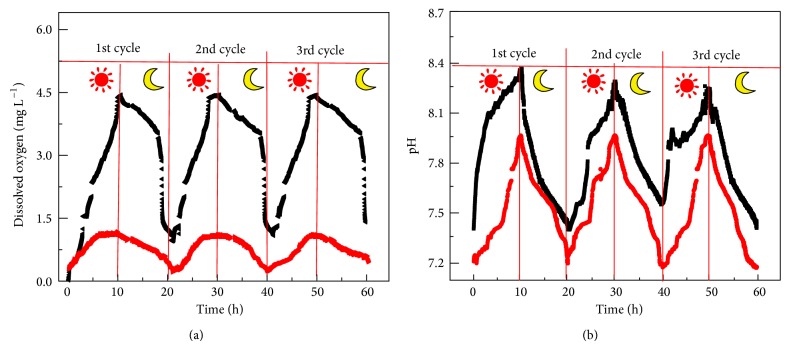
Changes in dissolved oxygen concentration (a) and pH (b) of the cathode electrolyte during illumination and dark circulation. (The symbols of moon and sun represent light and dark conditions, resp.). (Black line: CO_2_ was allowed to travel through the tube; red line: CO_2_ travel through the silicon tube was blocked.)

DO and pH in the cathode chamber are important parameters affecting the electricity generation in the MCC. DO concentration and pH were* in situ* determined using microelectrodes. As shown in Figure 5, the maximal concentration of DO reached 4.5 mg/L when the anode off gas was piped into the cathode and the illuminated cathode compartment. Meanwhile, the maximal concentration of DO reached 1.2 mg/L when the anode off gas was not piped into the illuminated cathode compartment. The unexpected oxygen production was believed to result from algal photosynthesis because oxygen concentration in the catholyte was in response to light illumination. As mentioned above, CO_2_ is required for photosynthesis by algae. Therefore, it is deduced that CO_2_ generated in the anode chamber had possibly entered the cathode chamber through a pathway other than the external pipe. In other words, it is believed that CO_2_ crossover through the Nafion membrane from the anode to the cathode compartment took place in the MCC. The phenomenon of CO_2_ crossover from the anode to the cathode through the Nafion membrane was previously confirmed in a direct methanol fuel cell [23], which supported our hypothesis.

The pH of the catholyte increased from 7.3 to 8.3 when the CO_2_ generated from the anode was piped into the cathode chamber under illumination, which was likely due to oxygen reduction. Note that the pH variation was well associated with the voltage output process during the light-on and -off circumstances. As previously reported, the MFC cathode reaction could elevate the pH of the catholyte to above 12 [24]. As expected, the same pH variation trend was observed when the CO_2_ was not piped into the cathode, further confirming occurrence of CO_2_ crossover from the anode to the cathode through the Nafion membrane in the constructed MCC.

### 3.4. Mechanisms and Implications

**Figure 6:**
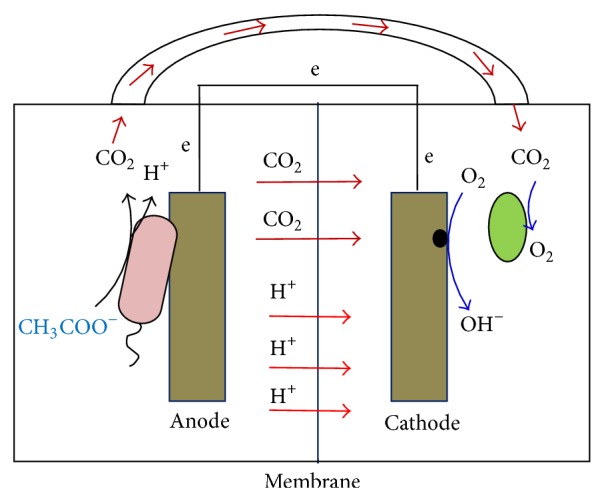
Schematic representation of CO_2_ transportation and anode and cathode reactions involved.

In general, three possible reactions take place in a photocathode chamber, including direct CO_2_ reduction, electron transfer through self-produced mediators, and reduction of oxygen generated through photosynthesis. However, Wang et al. [14] previously suggested that reduction of oxygen generated through photosynthesis was the major contributor to the high current generation in an MCC. Oxygen generation relied on the uptake of CO_2_ by algae. However, previous study had only considered the CO_2_ transportation through the pipe. Here, we showed that the cathodic photosynthesis still took place when the anodic gas was no longer piped into the catholyte. In this case, we deduced that CO_2_ crossover from the anode to the cathode through the Nafion membrane contributed to the photosynthetic oxygen generation (Figure 6). In general, the following main reactions occurred in our system.

Anode reaction is as follows:

(1) $$\begin{array}{c} {\text{CH}}_{3}{\text{COO}}^{-}+2{\text{H}}_{2}\text{O}⟶2{\text{CO}}_{2}+7{\text{H}}^{+}+8{\text{e}}^{-} \end{array}$$

Cathode reaction is as follows:

(2) nCO2+nH2O⟶CH2On+nO2

(3) $$\begin{array}{c} {\text{O}}_{2}+4{\text{e}}^{-}+4{\text{H}}^{+}⟶2{\text{H}}_{2}\text{O} \end{array}$$

(4) $$\begin{array}{c} {\text{O}}_{2}+2{\text{H}}_{2}\text{O}+4{\text{e}}^{-}⟶4{\text{OH}}^{-} \end{array}$$

Note that oxygen is generally considered to be reduced at the Pt surface in the cathode through reaction (3) [25]. However, alkalization of the catholyte was observed in the MCC, suggesting that oxygen was reduced to produce OH^−^ as a main product as shown in reaction (4) [26]. It should be noted that the theoretical potential of the ORR in reaction (4) is lower than that in reaction (3), representing a great potential loss because of the alkalization of the catholyte. As previously suggested, decreasing the pH value of the catholyte during the MCC operation can be one solution to achieve higher performance [27].

MFC has been considered as a sustainable technology for energy production and wastewater treatment. However, energy consumption was necessary to maintain MFC operations. Algal photosynthesis provides an option to eliminate or maintain minimum energy consumption in MFC technology by omitting aeration. Therefore, aeration was not required and the greenhouse gas (CO_2_) emitted from the anode chamber was self-sequestrated in the MCCs, making real green systems for energy generation. However, it is notable that energy production of the photosynthetic algal MFCs is currently quite low compared with conventional MFCs. Light intensity was proved to be one of the critical parameters in affecting their performance [16]. Other parameters such as reactor configurations, algal species, and electrode materials should be improved in the near future for targeting high-performance algal photo MFC systems.

## 4. Conclusion

In this study, an MCC was constructed and evaluated in terms of its power output. The results showed that the MCC was sensitive to light no matter the anode off gas was piped into the catholyte or not. Oxygen was produced and alkalization occurred in the cathode compartment, suggesting the occurrence of the photosynthetic reaction and oxygen reduction reactions under both conditions. We concluded that the CO_2_ crossover through Nafion membrane contributed to the oxygen production when the anode off gas was not piped into the cathode chamber. The results are expected to help further advance the MCC technology.
